# Achieving Strength and Toughness in Dual-Phase Mg-8Li Alloys Through Phase Structure Control and Composite Fracture

**DOI:** 10.3390/ma17235984

**Published:** 2024-12-06

**Authors:** Lei Zhao, Shuaipu Wang, Mingjian Wu, Chengxiang Liu, Zhilin Wu

**Affiliations:** School of Mechanical Engineering, Nanjing University of Science and Technology, Nanjing 210094, China; zhlei@njust.edu.cn (L.Z.); wang_sp2023@163.com (S.W.); wmj@njust.edu.cn (M.W.); pingfangyuan721@163.com (C.L.)

**Keywords:** Mg-Li alloy, strengthening and toughening, heterostructured materials, dual-phase

## Abstract

The rising industrial demand for ultra-lightweight materials with exceptional strength and toughness has intensified interest in dual-phase Mg-Li alloys due to their low density and high specific strength. While much of the research on Mg-Li alloys has concentrated on conventional strengthening methods, such as grain refinement and solid-solution strengthening, overcoming the challenge of plastic deformation compatibility between the α- and β-phases remains unresolved. This study focuses on Mg-8Li binary alloy, systematically investigating the impact of rolling deformation temperature and strain on the phase structures. A detailed analysis of fracture behavior reveals a novel brittle–tough composite fracture control strategy that enhances both strength and toughness simultaneously. This work advances the understanding of phase structure control and its role in strengthening and toughening mechanisms, offering critical insights for the development of next-generation dual-phase magnesium alloys.

## 1. Introduction

As the demand for lightweight materials rises in the aerospace and automotive industries, there is a growing need for high-performance, lightweight alloys [1,2,3]. Magnesium alloys, renowned for their low density, have garnered considerable attention for their potential in enhancing strength and toughness [4,5]. However, the hexagonal close-packed (HCP) crystal structure of magnesium restricts the number of slip systems activated at room temperature, making it difficult to achieve stable and uniform plastic deformation [6,7,8]. Additionally, the well-known trade-off between strength and ductility in metals remains a significant challenge in magnesium alloys, as conventional strengthening methods often reduce ductility [9,10,11,12]. This challenge is further compounded by the inherently low plasticity of magnesium alloys, complicating efforts to simultaneously attain high strength and toughness [13,14].

To address these limitations, one of the promising strategies for optimizing the balance between strength and ductility is the introduction of dual-phase heterostructures in magnesium alloys [15,16,17,18]. Among these, magnesium–lithium alloys—composed of both α- and β-phases when lithium content ranges between 5.7% and 10.3%—have emerged as a leading example [19,20]. The β-phase, with its body-centered cubic (BCC) structure, offsets the limited slip systems of the α-phase’s HCP structure. Furthermore, the plastic mismatch between the phases during deformation generates geometrically necessary dislocations at the phase interface [21,22], enhancing dislocation density and activating additional slip systems. For example, Xie et al. utilized in situ neutron diffraction to investigate the deformation mechanisms in dual-phase Mg-Li alloys, identifying early yielding and hardening in the β-phase, along with the sequential activation of basal and prismatic slip in the α-phase, as key factors enhancing ductility [23]. Zhou et al. developed a bimodal-structured Mg-Li alloy, where the primary strengthening mechanisms include grain boundary strengthening, precipitation strengthening, and dislocation strengthening [24]. Klu et al. successfully fabricated a dual-phase Mg-9Li alloy that demonstrates an excellent balance of strength and ductility. The high strength is attributed to grain refinement and dislocation strengthening, while the enhanced ductility is primarily due to the contributions from texture [25].

The interaction between the soft and hard regions in dual-phase alloys also plays a pivotal role. When subjected to stress, the phases exhibit different capacities for strain accommodation, resulting in significant strain gradients at the interface, which are necessary to maintain deformation compatibility [21,22]. Li et al. demonstrated that variations in α-phase morphology within dual-phase Mg-Li alloys alter the interface area, thereby influencing mechanical behavior [26]. Their findings, supported by experimental and computational data, revealed that reducing the strength disparity between phases via hetero-deformation-induced (HDI) stress enhances coordinated deformation [27]. These insights underscore the importance of phase coordination during service deformation and emphasize the need for effective structural control methods. In this work, the Mg-8Li binary alloy was chosen to systematically investigate the effects of rolling deformation temperature and strain on the α- and β-phases. The fracture behavior and underlying mechanisms were analyzed, with brittle–tough composite fracture control proposed as a strategy to simultaneously enhance strength and toughness. This work explores phase structure control strategies and elucidates the strengthening–toughening mechanisms, offering both theoretical and technical foundations for the advancement of dual-phase magnesium alloys.

## 2. Materials and Methods

Dual-phase Mg-Li alloys were used for a systematic investigation of their strengthening and toughening mechanisms. The chemical composition of the Mg-8Li alloy was determined using an inductively coupled plasma optical emission spectrometer ICAP-Pro (Thermo Fisher Scientific Company, Waltham, MA, USA), with the results cataloged in Table 1. Drawing from the Mg-Li binary phase diagram, we employed a high-temperature solution treatment aimed at homogenizing phase distribution and eliminating solute segregation. Additionally, we optimized the solution treatment conditions by increasing temperature and extending the treatment duration to reduce the presence of secondary phases from smelting impurities. The as-received alloys underwent homogenization at 550 °C for 12 h (T4), followed by water quenching to room temperature, producing a dual-phase microstructure. To prevent surface oxidation during the process, the chamber was evacuated to a vacuum of 3 × 10^−3^ Pa and filled with high-purity argon gas at a pressure of ~200 Pa for protection. The initial thickness of the rolled plates was 2.5 mm. Surfaces were polished, and edges chamfered to minimize edge cracking. The room temperature rolling samples were rolled directly, while the cryogenic and warm rolling samples were prepared in insulated containers with liquid nitrogen and in a muffle furnace (Nabertherm Company, Lilienthal, Germany) preheated to 300 °C, respectively, with a 10 min holding period. For each sample type, the rolling process reduced thickness by 0.1 mm per pass, achieving the final thicknesses of 2 mm, 1.5 mm, and 1 mm, corresponding to the strain levels of 20%, 40%, and 60%, respectively.

Quasi-static uniaxial tensile tests were conducted using a w+b LTM-20 kN mechanical testing machine (Walter+Bai Company, Löhningen, Switzerland) at a strain rate of 1 × 10^−3^ s^−1^ at room temperature. The tensile specimens were dog-bone shaped with a gauge length of 10 mm, width of 2.5 mm, and thickness of 1 mm. During the tensile deformation, an Epsilon extensometer with a gauge length of 5 mm was used. The length direction was parallel to the rolling direction. To ensure data accuracy, three specimens for each heat treatment condition were tested, and the average results were reported.

Specimens were prepared for a microstructural analysis by grinding with 320 to 2000 grit sandpaper and polishing with a woolen cloth and 0.3 μm Al_2_O_3_ suspension. Due to the reactivity between Mg-Li alloys and water, the polished samples required no additional etching for observation via optical microscopy (OM) and scanning electron microscopy (SEM). Microstructural evolution was examined using an FEI Quanta 250F SEM (FEI Compang, Hillsboro, OR, USA). Transmission electron microscopy (TEM) samples were sectioned perpendicular to the deformation direction, polished to ∼25 μm thickness, and perforated by ion milling at −30 °C with a low-angle (<3.5°) and low-energy (<3 keV) ion beam. The TEM analysis was conducted on an FEI-Tecnai G2 20 LaB_6_ microscope operated at 200 kV.

## 3. Results

**Figure 1:**
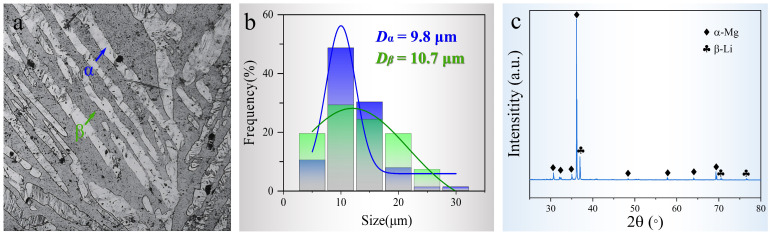
Microstructure of solid solution-treated Mg-8Li alloy. (**a**) Optical image showing a lamellar structure stacked by α- and β-phases; (**b**) size distribution of the lamellae thickness; (**c**) XRD pattern of the dual-phase structure.

The starting material investigated in this study is a solid solution-treated (T4) Mg-8Li alloy. As shown in Figure 1a, the 8% Li content places the alloy within the two-phase region of Mg-Li systems, resulting in the coexistence of both α-phase and β-phase after the solution treatment, characterized by a lamellar microstructure with alternating light and dark gray layers. This morphology is characteristic of Mg-Li dual-phase alloys. Notably, the β-phase region is not a pure β-phase but comprises both β-phase and finely dispersed α-phase. As shown in Figure 1b, the statistical measurements reveal an average lamellar thickness of approximately 10 µm for both phases, with the α-phase displaying greater uniformity in thickness compared to the more variable β-phase. An X-ray diffraction (XRD) analysis (Figure 1c) further corroborates the phase constitution, with distinguishable diffraction peaks for both α- and β-phases. The higher relative intensity of the α-phase peaks indicates that the α-phase predominates within the Mg-8Li alloy.

**Figure 2:**
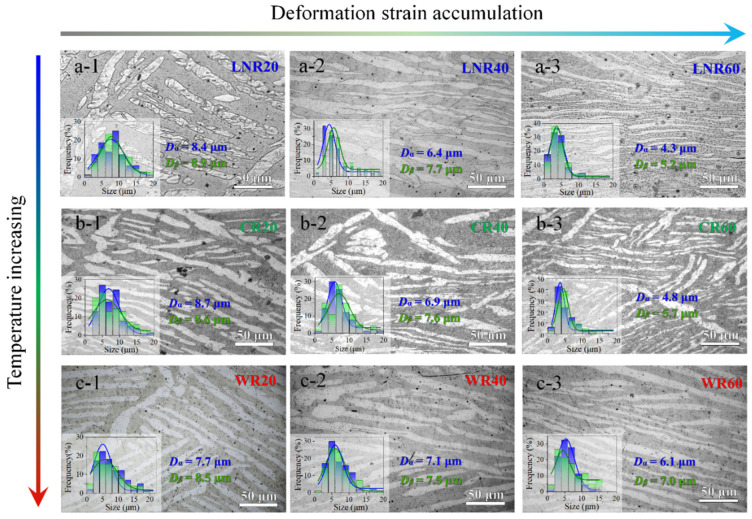
Effect of temperature and rolling reduction on the microstructure evolution of the Mg-8Li alloy. (**a-1**–**a-3**) Optical micrography of the cryogenic-rolled sample with reductions of 20%, 40%, and 60%, respectively. (**b-1**–**b-3**) Optical micrography of the room temperature-rolled sample with reductions of 20%, 40%, and 60%, respectively. (**c-1**–**c-3**) Optical micrography of the 300 °C warm-rolled sample with reductions of 20%, 40%, and 60%, respectively.

**Figure 3:**
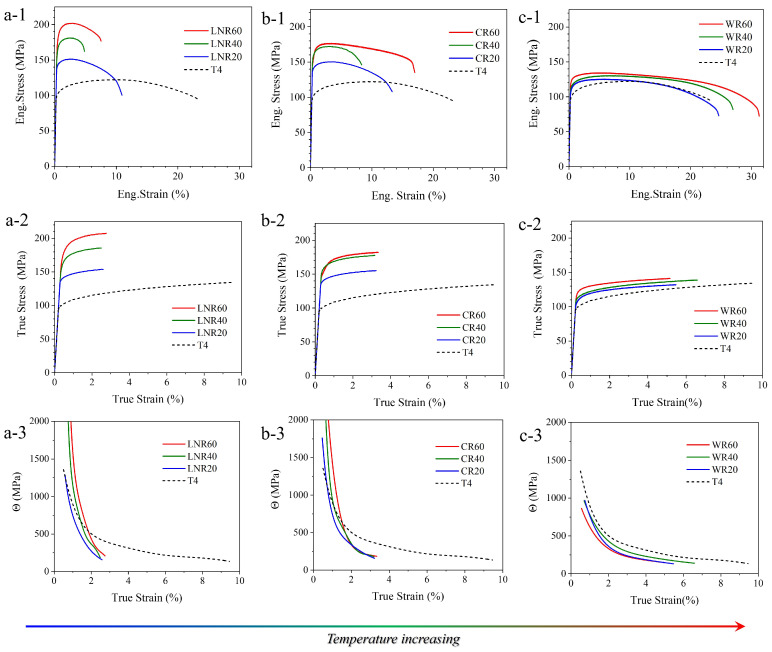
Effect of deformation temperature and rolling strain on the tensile mechanical properties of the Mg-8Li alloy. (**a-1**–**a-3**) The engineering stress–strain, true stress–strain, and strain hardening curves of the cryogenic-rolled samples. (**b-1**–**b-3**) The engineering stress–strain, true stress–strain, and strain hardening curves of the room temperature-rolled samples. (**c-1**–**c-3**) The engineering stress–strain, true stress–strain, and strain hardening curves of the 300 °C warm-rolled samples.

Dual-phase materials inherently possess a heterostructured nature, with mechanical properties that can differ significantly between phases due to variations in crystal structure [28,29]. Previous research has revealed the crucial influence of deformation temperature on microstructural evolution during the plastic deformation of such dual-phase systems. Temperature regulation is key in facilitating coordinated deformation between the two phases, profoundly affecting lamellar structure refinement [30]. Here, we subjected the Mg-8Li alloy to rolling deformation at liquid nitrogen temperature, room temperature, and 300 °C, respectively. As shown in Figure 2(a-1–a-3), rolling at liquid nitrogen temperature with 20%, 40%, and 60% reduction leads to increasingly uniform lamellar alignment, with the lamellar thickness reduced from 10 µm to 5 µm. Notably, the α-phase exhibits more pronounced refinement than the β-phase at this temperature, though no shear bands—indicative of phase-coordinated deformation—were observed. Figure 2(b-1–b-3) illustrate the microstructural evolution at room temperature, where lamellar refinement is comparable to that observed at liquid nitrogen temperature. However, the alignment effect is slightly weaker, and shear bands begin to form between lamellae, a feature typically associated with phase-coordinated deformation in duplex systems [31]. At 300 °C (Figure 2(c-1–c-3)), lamellar refinement decreases slightly, likely due to increased defect recovery within grains at elevated temperatures. This indicates a lower density of deformation-induced defects, which typically enhances the plasticity and toughness of materials [32].

The mechanical properties of the Mg-8Li alloy sheets vary notably across different rolling temperatures. Figure 3(a-1–a-3) shows that cryogenic rolling produces the most pronounced increase in strength, with the yield strength rising from 100 MPa to over 200 MPa as deformation advances. However, this enhancement comes at the cost of ductility, with fracture elongation decreasing from 22% to less than 10%. Strength continues to rise with further deformation, while elongation decreases up to 40% deformation, followed by a slight recovery. The true stress–strain curves reveal that the liquid nitrogen-rolled material exhibits uniform plastic deformation, constrained to approximately 2.5% (Figure 3(a-2)). Work hardening curves indicate a rapid decline in the work hardening rates of the LNR20, LNR40, and LNR80 samples, resulting in an early onset of deformation instability (Figure 3a-3). By contrast, as shown in Figure 3(b-1–b-3), room temperature rolling shows diminishing strength gains beyond 40% deformation, while fracture elongation improves significantly, following a trend similar to that observed under cryogenic rolling. The warm-rolled samples, on the other hand, exhibit minimal strength enhancement; instead, increasing rolling reduction leads to a gradual rise in fracture elongation and uniform elongation, ultimately exceeding 30% and 5%, respectively (Figure 3(c-1–c-3)). These findings highlight a distinct trade-off between strength and ductility that varies with the thermal conditions during processing.

**Figure 4:**
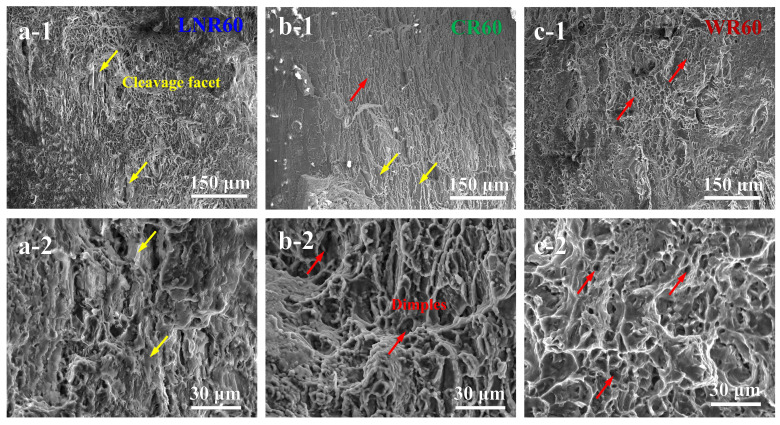
Effect of deformation temperature and rolling strain on the fracture behavior of the Mg-8Li alloy. (**a-1**,**a-2**) SEM images of the cryogenically rolled samples, showing micro-voids on the fracture surface. (**b-1**,**b-2**) SEM image of the room temperature-rolled samples with a combination of micro-voids and dimples. (**c-1**,**c-2**) SEM images of the warm-rolled samples revealing ductile fracture characterized by dimples.

During cryogenic deformation, the dual-phase Mg-Li alloys exhibited significant strain hardening, indicating that dislocation activity dominates at this temperature. As the temperature increases, the strain hardening behavior gradually weakens, but the total elongation after necking increases. This phenomenon may be related to the strain partitioning between the α- and β-phases during tensile testing. To investigate further, we conducted a microscopic analysis of the tensile fracture surfaces. As shown in Figure 4(a-1,a-2), the fracture of the cryogenic-rolled sample was predominantly brittle, with distinct micro-voids observable as a typical fracture feature, as marked by yellow arrows. The plastic deformation at liquid nitrogen temperature led to significant work hardening, but the uniform elongation was less than 5%. This behavior is consistent with the deformation of most magnesium alloys dominated by basal dislocation slip [33]. The reason is that extensive basal slip in the HCP-structured α-phase grains easily triggers strain localization, leading to necking and deformation instability [32]. In contrast, the fracture surface of the hot-rolled sample predominantly featured dimples, as marked by the red arrows in Figure 4(c-1,c-2). Although this sample exhibited the characteristics of ductile fracture, its work hardening capacity was quite poor, even lower than that of the cryogenic-rolled sample. However, despite the necking, it still maintained up to 30% elongation, suggesting that the necking was not caused by dislocation pile-up and that strain localization was minimal. The reason for this phenomenon is related to the deformation of the β-phase with a BCC crystal structure [34,35]. The β-phase easily undergoes intergranular deformation, and within its grains, dislocations tend to cross-slip, significantly reducing dislocation pile-up and entanglement [20]. As a result, the material tends to exhibit low strength but high elongation [36]. Therefore, the α- and β-phases each possess distinct plastic deformation characteristics. If their deformation could be coordinated, the material system would benefit from both high strength due to work hardening and high toughness due to non-localized strain. As shown in Figure 4(b-1,b-2), the fracture surface of the room temperature-rolled sample with 60% reduction is a composite fracture featuring both micro-voids and dimples. This indicates that room temperature rolling deformation helps harness the advantages of both phases in the duplex structure, enabling the material to achieve both high strength and high toughness.

**Figure 5:**
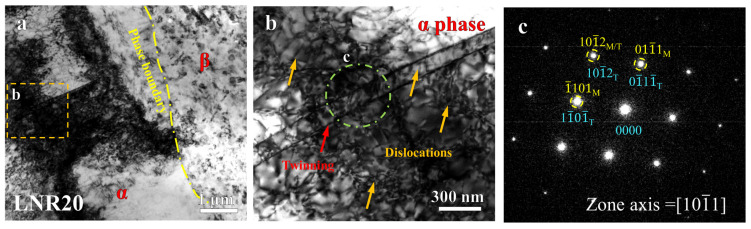
Detailed microstructure of the Mg-8Li alloys subjected to 20% of cryogenic rolling. (**a**) Bright-field TEM image near a phase boundary between the α- and β-phases. (**b**) Enlarged image of the orange dash line box in (**a**). (**c**) Selected area diffraction pattern in the $\left[10\bar{1}1\right]$ zone axis.

Through the above analysis of mechanical properties and fracture behavior, we propose that the pronounced strain hardening effect observed in the samples deformed at cryogenic temperatures is primarily attributed to plastic deformation mechanisms initiated within the α-phase grains. To explore this further, we employed TEM to examine the microstructure of a 20% cold-rolled sample. As shown in Figure 5a, a distinct phase boundary between the α- and β-phases is found, delineated by the yellow dashed line, which exhibits differences in grain size. The α-phase exhibits a high dislocation density, obstructed by lamellar structures on the order of tens of nanometers, resulting in local dislocation pile-up. Figure 5b provides a magnified view of the lamellar structure, revealing regular interfaces where dislocation accumulation is particularly pronounced. Selected area electron diffraction was utilized to analyze the orientation of these interfaces, with the corresponding diffraction pattern displayed in Figure 5c. The observed zone axis is $\left[10\bar{1}1\right]$, a choice that, while not the lowest-index zone axis, is commonly employed to assess whether the lamellar structure in magnesium alloys arises from tensile twinning, as indicated by the presence of $\left(10\bar{1}2\right)$ diffraction spots [37,38]. It is clear that during the initial deformation stages of the liquid nitrogen cold-rolled samples, deformation twins were introduced into the α-phase. These twins serve as nucleation sites for dislocation emission [39,40] while simultaneously impeding dislocation slip, thereby enhancing the work hardening response.

**Figure 6:**
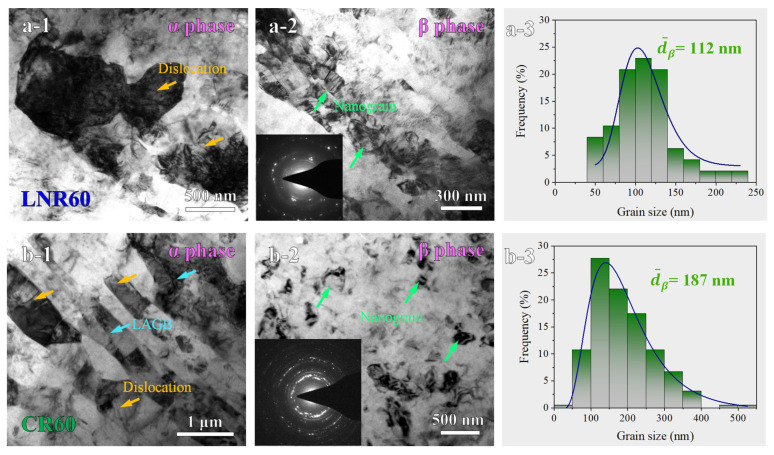
Comparison of the detailed microstructure of the LNR60 and CR60 samples. (**a-1**) Bright-field TEM image of the α-phase grains. (**a-2**,**a-3**) TEM image, SAED, and the corresponding grain size of the β-phase grains. (**b-1**) Bright-field TEM image of the α-phase grains. (**b-2**,**b-3**) TEM image, SAED, and the corresponding grain size distribution of the β-phase grains.

An essential question that requires clarification is why room temperature rolling promotes cooperative deformation between the α- and β-phases, while cryogenic rolling does not. Figure 6(a-1,a-2) show the TEM structural analyses of the α- and β-phase regions in the Mg-Li alloy rolled at liquid nitrogen temperature. The α-phase exhibits a nearly equiaxed grain structure with an average grain size of ~500 nm and a high dislocation density. In contrast, the β-phase is characterized by a lamellar structure composed of nanocrystals. Figure 6(a-3) shows that the statistical analysis of the β-phase grain size has an average size of 112 nm, indicating that the α-phase is roughly five times larger than the β-phase in the liquid nitrogen-rolled sample. After rolling at room temperature, however, the α-phase transitions to a lamellar structure with a thickness of about 200–300 nm, as illustrated in Figure 6(b-1). Figure 6(b-2,b-3) reveal that the average grain size of the β-phase increases to 187 nm, resulting in comparable grain sizes for the α- and β-phases post room temperature rolling. Although the Hall–Petch relationship is often employed to describe the dependence of yield strength on grain size, its application to dual-phase materials is limited due to differing K values for the α- and β-phases. In nanocrystalline materials, where the reduced grain size constrains the number of dislocations that can activate within each grain, Equation (1) provides a more accurate assessment of dislocation activation difficulty:

(1) τ=Gb/L

where *G* is the shear modulus, *b* is the Burgers vector, and *L* is the characteristic length of a Frank–Read source. In nanocrystalline and ultrafine-grained materials, the critical shear stress *τ* for dislocation activation is strongly influenced by L, which can be approximated as the grain size in nanocrystalline systems [41,42,43]. Consequently, the τ value for the β-phase in the liquid nitrogen-rolled samples is twice that of the room temperature-rolled samples, while the *τ* value for the α-phase is only one-fifth that of the room temperature-rolled samples. This indicates that dislocation activation in the α-phase is considerably more facile in liquid nitrogen-rolled samples, leading to deformation being largely restricted to the α-phase. In contrast, the conditions in the room temperature-rolled samples facilitate cooperative deformation between the α- and β-phases.

## 4. Discussion

### 4.1. Strategy of Phase Structure Control

The defining microstructural feature of dual-phase Mg-Li alloys, in contrast to conventional magnesium alloys, is the coexistence of the HCP-structured α-phase and the BCC-structured β-phase. The precise control of the Li content during the alloying process is required to establish the desired phase ratio [16]. Once set, the thickness of these two-phase regions can be further manipulated through rolling and plastic deformation [30,44,45]. Two key factors must be considered when controlling this structure: The first is the strain of deformation. Due to the differing yield strengths of the two phases, grain refinement during plastic deformation occurs asynchronously. In this study, a temporary decrease in total elongation was noted at a 40% reduction in thickness, but significant improvement was observed at a 60% reduction in rolling. This indicates that increasing the degree of deformation helps synchronize the deformation between the two phases, optimizing mechanical performance. The second is deformation temperature. Mg-Li alloys exhibit excellent workability over a wide range of temperatures, from cryogenic (liquid nitrogen) to warm rolling at 300 °C. However, careful control is needed to avoid excessively high temperatures, which accelerate dislocation recovery and reduce defect density, limiting grain refinement and material strengthening. Conversely, very low temperatures, such as 77 K, can lead to the disproportionate refinement of the β-phase relative to the α-phase, concentrating strain in the α-phase and increasing the risk of premature necking and fracture during service. Thus, the successful regulation of the phase structure in dual-phase Mg-Li alloys hinges on the coordination of the two phases during plastic deformation and their behavior in service. A well-devised microstructural control strategy must account for both phase compatibility and the sequence of deformation, ensuring that the alloy’s mechanical properties are optimized for long-term performance.

### 4.2. Mechanism for Achieving High Strength and Toughness Through Phase Coordination

The mechanical property results from this study reveal that the samples deformed at room temperature exhibit not only increased strength but also retain significant total elongation. This enhancement is attributed to the optimized utilization of both the α- and β-phases. The HCP-structured α-phase contributes to work hardening by accumulating dislocations during deformation, thereby enhancing both strength and strain hardening capacity. In contrast, the BCC-structured β-phase, with its more open crystal structure, facilitates the easy cross-slip of dislocations and promotes intergranular deformation. The participation of the β-phase in plastic deformation enables the material to resist premature necking and instability after reaching its tensile strength, thus maintaining ductility. Achieving both high strength and toughness in dual-phase Mg-Li alloys depends on maximizing the cooperative deformation between the α- and β-phases. The α-phase provides the necessary strain hardening rate, while the β-phase suppresses strain localization during necking. This synergistic interaction between the phases results in Mg-Li alloys that exhibit an ideal balance of strength and toughness, making them promising materials for applications requiring both properties.

## 5. Conclusions

In this work, we proposed the use of phase structure control as the primary means to achieve brittle–ductile composite fracture in dual-phase Mg-Li alloys, thereby simultaneously enhancing the material’s strength and plasticity. The main findings are as follows:

(1) Preventing strain localization within either the α- or β-phase is essential for enhancing toughness. When manipulating the phase structure of the dual-phase Mg-8Li alloy through rolling, it is important to slightly increase the grain size of the ultrafine β-phase. Additionally, during rolling deformation, the α-phase’s capacity to accommodate strain should be optimized by controlling its grain morphology to a lamellar structure, which facilitates a reduction in the gap of critical shear stress required for dislocation activation in both phases.
(2) Cryogenic rolling increases strength but reduces ductility, while room temperature rolling balances both. The warm-rolled samples show minimal strength gain but higher elongation. The microscopic analysis reveals brittle fracture and work hardening at cryogenic temperatures, while warm rolling shows ductile behavior with less dislocation pile-up. Room temperature rolling combines brittle and ductile features, enhancing both strength and toughness by leveraging the dual-phase structure.
(3) Strain hardening in the cryogenic-rolled Mg-8Li alloys is driven by dislocation activity in the α-phase, where high dislocation density and lamellar structures cause pile-up. Cryogenic rolling also introduces deformation twins that enhance work hardening. Room temperature rolling, however, enables cooperative deformation between the α- and β-phases due to similar grain sizes. The variation in grain size and dislocation activation explains the different deformation behaviors at each temperature.

## Figures and Tables

**Table 1:** Chemical compositions of Mg-Li alloys (wt.%).

	Chemical Composition (wt.%)	
	Mg	Li
Mg-8Li	Bal.	8.36

## Data Availability

The raw data supporting the conclusions of this article will be made available by the authors upon request.
